# A questionnaire survey regarding the support needed by *Yogo* teachers to take care of students suspected of having eating disorders (second report)

**DOI:** 10.1186/s13030-016-0079-z

**Published:** 2016-09-29

**Authors:** Kaoru Seike, Michiko Nakazato, Hisashi Hanazawa, Toshiyuki Ohtani, Tomihisa Niitsu, Shin-ichi Ishikawa, Atsuko Ayabe, Ryoko Otani, Kentaro Kawabe, Fumie Horiuchi, Shizuo Takamiya, Ryoichi Sakuta

**Affiliations:** 1 United Graduate School of Child Development, Osaka University, 2 Yamadaoka, Suita-city, Osaka Prefecture 565-0871 Japan; 2 Research Center for Child Mental Development Chiba University, 1-8-1 Inohana, Chuo-ku, Chiba-city, Chiba Prefecture 260-8670 Japan; 3 Faculty of Education, Chiba University, 1-33 Yayoi, Inage-ku, Chiba-city, Chiba Prefecture 263-0022 Japan; 4 Safety and Health Organization, Chiba University, 1-33 Yayoi, Inage-ku, Chiba-city, Chiba Prefecture 263-0022 Japan; 5 Department of Psychiatry, Graduate School of Medicine, Chiba University, 1-8-1 Inohana, Chuo-ku, Chiba-city, Chiba Prefecture 260-8670 Japan; 6 Department of Psychiatry, Nishi-Kobe Medical Center, Kobe, 5-7-1 Koujidai, Nishi-ku, Kobe-city, Hyogo Prefecture 651-2273 Japan; 7 Kishibe Psychiatry Clinic, 1-24-1 Kishibe Minami, Suita-city, Osaka Prefecture 564-0011 Japan; 8 Center for Child Development and Psychosomatic Medicine, Dokkyo Medical University Koshigaya Hospital, 2-1-50 Minami-koshigaya, Koshigaya-city, Saitama Prefecture 343-0845 Japan; 9 Department of Neuropsychiatry, Ehime University Graduate School of Medicine, Shitsukawa Toon-City, Ehime Prefecture 791-0204 Japan; 10 Center for Child Health, Behavior and Development, Ehime University Hospital, Shitsukawa Toon-City, Ehime Prefecture 791-0204 Japan; 11 Takamiya Mental Clinic, Akashi, Marunouchi Bldg 5F, 2-5-2 Marunouchi, Akashi-city, Hyogo Prefecture 673-0016 Japan

**Keywords:** DSM-5, Eating disorder, Support, Surveillance, *Yogo* teacher

## Abstract

**Background:**

The lowering of the age of onset and chronicity have been key problems related to eating disorders (EDs). As the proportion of teens in the estimated onset ages has increased, it has become important to detect students with EDs and to clarify how they can be supported. Though epidemiological surveys of *Yogo* teachers (school nurse/health science teachers) have been conducted to inquire about the number of such students, none of these were done according to ED type based on DSM-5. Thus, we conducted a wide area survey in Japan with the goal of proposing a better framework of support for *Yogo* teachers in their efforts to care for students with EDs.

**Methods:**

A questionnaire survey organized by ED type (based on DSM-5) was administered to *Yogo* teachers working at elementary/junior high/senior high/special needs schools in four prefectures of Japan in 2015, and 1,886 responses were obtained. Based on the results, the encounter rates (the proportion of *Yogo* teachers who had encountered a student with an ED) were calculated, and factors that could affect the rates were examined by logistic regression analysis.

**Results:**

The order of the encounter rates of the ED types was as follows: Anorexia Nervosa (AN) > Bulimia Nervosa (BN) > Avoidant/Restrictive Food Intake Disorder (ARFID) > Binge Eating Disorder (BED) > Others. The factors significantly affecting the rates were “location, school type, number of students, experience years, and AN knowledge” for AN, “school type, experience years, and BN knowledge” for BN, “school type, experience years, and BED knowledge” for BED, “location, experience years, and ARFID knowledge” for ARFID, and “school type, experience years, and Others knowledge” for Others.

**Conclusions:**

Because the encounter rate of AN was the highest, providing support for AN would be the most effective. Moreover, one factor that affected the encounter rate of all ED types was ED knowledge. In addition to this, senior high schools had the highest encounter rates for AN, BN and BED, and special needs schools had the highest rates for Others. These findings imply that, in order to detect and support ED students at an early stage, it is necessary to offer knowledge of the most prevalent ED types to *Yogo* teachers at the corresponding school type.

## Background

The lowering of the age of onset and chronicity have been found in recent years to be key problems related to eating disorders (EDs) [1–9]. Children with EDs are likely to fall into physical crises and develop growth disorders [10–13]. It is necessary to diagnose them and intervene at an early stage. Children with EDs, especially Anorexia Nervosa (AN), strongly resist treatment, and often do not visit medical institutions. Therefore, actual condition surveys are believed to be necessary. Moreover, it is important to identify students suspected of having EDs early and to clarify the way they are supported in school, because the proportion of teens at the estimated onset age of ED has increased year by year [14].

It is important to determine how many ED students are present in school in order to provide early support. Although school physicians do medical check-ups and recommend that students suspected of having EDs visit medical institutions based on Japanese law, half to one-third of students with AN do not follow the advice [15]. Therefore, school staff members have been a focus of attention in recent years, as they are in the best position to identify the early warning signs of EDs and to support ED students [16]. *Yogo* teachers are unique teachers in Japan. They not only take care of students in their capacity as school nurses, but also are in charge of health education. They stay at school on weekdays, teach courses on adolescent health and have contact with parents. They are not required to have nursing licenses, although some of them do [15]. Actually, epidemiological surveys of *Yogo* teachers have achieved fruitful results in recent years, and many of them reported the lowering of the age of onset of EDs [17–19].

While many epidemiological actual condition surveys of AN have been conducted, surveys of other ED types have rarely been done in Japan [18] (although some have been done outside Japan [20–26]). Moreover, all of the Japanese studies were based on the diagnostic criteria in DSM-IV [15, 17–19], and no survey by ED type has been done based on the new diagnostic criteria in DSM-5.

The ED types described in DSM-5 are AN, Bulimia Nervosa (BN), Binge Eating Disorder (BED), Avoidant/Restrictive Food Intake Disorder (ARFID), and other types of EDs such as rumination disorder or pica (Others).

The American Psychiatric Association revised the DSM diagnosis criteria in 2013, and “Eating Disorders” and “Feeding and Eating Disorders in Infancy or Early Childhood” in DSM-IV were integrated into a new diagnosis category, “Eating Behavior Disorders and Eating Disorders.” This change can be attributed to the fact that there was no need to separate infancy or early childhood from other ages, as it was widely recognized that EDs, previously believed to emerge mainly in infancy or early childhood, actually emerged at other ages too. In addition, the prevalence rates of EDs were reported to be higher in DSM-5 than DSM-IV-TR, for DSM-5 has an increased number of symptom names and more flexible diagnostic criteria [22–33]. These facts mean that a new survey by ED type based on DSM-5 is necessary.

Our previous study surveyed the encounter rate to effectively support *Yogo* teachers in giving early support to students with EDs [34]. However, the results of that study could be biased since the survey subjects were limited to Chiba prefecture, Japan. Moreover, our results were problematic in that the confidence intervals of the estimated proportions were long, owing to the small sample sizes for special needs schools and Others. (Special needs schools are institutes for educating children flexibly according to their conditions of dysfunction.)

In order to solve these problems, we chose samples from a wider area of Japan and surveyed the encounter rate by ED type. In addition, we surveyed for factors that could affect the rates in order to enhance the effectiveness of support for children with EDs. In other words, our objectives were to conduct a questionnaire survey of *Yogo* teachers in four prefectures, calculate the encounter rate by ED type (based on DSM-5), and examine the relations between the rates and location, school type, number of students, years of experience as a *Yogo* teacher, nursing experience, and ED knowledge.

## Methods

### Survey participants

The survey participants were *Yogo* teachers working at elementary, junior high, senior high, and special needs schools in Chiba prefecture, Saitama prefecture, Hyogo prefecture, and Ehime prefecture.

### Demographics of the prefectures

**Fig. 1:**
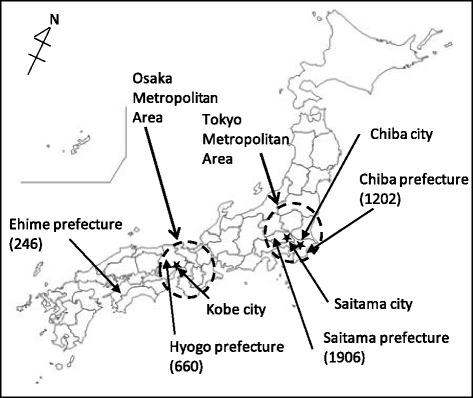
Characteristics of survey areas. Note) Population densities were calculated from data by [42] and [43]. : government‐ordinance‐designated city

Chiba, Saitama, Hyogo and Ehime are administrative divisions (prefectures) of Japan. Chiba and Saitama, prefectures in the Tokyo metropolitan area, had population densities of 1,202 and 1,906 people/km^2^, respectively, at the time of the study. Hyogo, a prefecture located in the Osaka metropolitan area, had 660 people/km^2^. Ehime, a prefecture located to the west of Osaka, had 246 people/km^2^. There are three government-ordinance-designated cities; Chiba, Saitama, and Kobe; - in these three prefectures. A government-ordinance-designated city is a city designated under Article 252, Section 19 of the Local Autonomy Law, and is a regional core with a population of more than 0.5 million that can be regarded as an urban area (Fig. 1). We classified the areas *Yogo* teachers worked in as “government-ordinance-designated cities” and “other areas”. Because there are no cities larger than government-ordinance-designated cities in these prefectures, “government-ordinance-designated cities” and “other areas” are roughly equal to “urban” and “rural” areas.

**Fig. 2:**
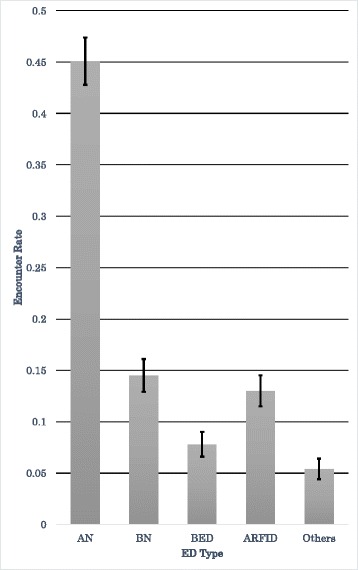
Encounter rates for each ED type (95 % CI)

### Study procedures

The questionnaire used was developed by an early detection working group committee. It consists of sections asking about the attributes of *Yogo* teachers, the features of their schools, and their experience encountering students with an ED, as well as a free description section for the respondents and an explanation of DSM-5.

The attribute section asked about the age, gender, nursing experience, years of experience as a *Yogo* teacher, and school type (elementary/junior high/senior high/special needs school). The section on the features of the school asked about the gender type (boys’/girls’/coeducational school), the number of students, and the location of the school she/he worked at. The section on the respondent’s knowledge of each ED type asked about their amount of knowledge, using a four-point scale (know well/know roughly/do not know much/do not know anything). The encounter rate section asked if the respondent had encountered students with an ED, by ED type (yes/no). In the free description section, the respondent could freely write impressions or requests regarding the survey. The explanation of DSM-5 described each ED type based on the DSM-5 diagnosis criteria; this section was added because not all *Yogo* teachers were familiar with these new criteria.

In Chiba prefecture, the questionnaires were distributed during a *Yogo* teacher seminar. In the other prefectures, they were mailed to elementary/junior high/senior high/special needs schools and collected by mail.

The results for the four prefectures were combined, and two kinds of analyses were conducted to fulfill our objectives.

1. The proportion of *Yogo* teachers who had encountered students with the various ED (encounter rate). (The encounter rate for each type of ED was calculated by dividing the number of *Yogo* teachers who had encountered each type by the total number of *Yogo* teachers who submitted the survey. Note that the encounter rate is not a prevalence rate, but the proportion of *Yogo* teachers who had the experience of taking care of or meeting students with EDs.)
2. Logistic regression analyses were performed with location, school type, number of students, years of experience as a *Yogo* teacher, nurse experience, and knowledge of ED as explanatory variables and experience encountering students with the various ED (yes/no) as a response variable.

Note that the total number of responses includes missing values. The statistical analysis software SPSS Ver. 21.0 (IBM, Tokyo, Japan) and the statistical power analysis software G*POWER Ver. 3.1.9.2 (University of Dusseldorf, Germany) were used for the analyses.

## Results and discussion

### Demographics of participants

The questionnaire surveys were conducted in 2015. First, for Chiba prefecture, a survey of 1,272 *Yogo* teachers was done at a Chiba prefecture *Yogo* teacher seminar in January. For the other prefectures, questionnaires were sent to the schools (614 in Hyogo in February, 492 in Ehime in February, and 1,301 in Saitama in June).

For Chiba, 661 responses were obtained (the effective response rate was 52.0 %). For Hyogo, 358 responses were received (58.3 %), Ehime had 362 (73.6 %) and Saitama had 503 (38.7 %). Combining these, the total sample size was 1,886.

G*POWER was used to verify the appropriateness of this sample size. By assuming the test method to be two-sided, the distribution to be normal, the significance level to be .05, the test power to be .80, and the odds ratio to be 1.3 in the logistic regression analysis, the required sample size was calculated to be 721. Moreover, the previous surveys of *Yogo* teachers reported sample sizes of 150 [18] and 391 [19]. Therefore, we judged our sample size to be large enough.

Tables 1 and 2 show the basic statistics regarding the attributes of *Yogo* teachers and their amount of ED knowledge. More than half (80.6 %) worked outside government-ordinance-designated cities (i.e., other areas) and about 19.4 % worked in government-ordinance-designated cities. More than half (56.0 %) worked at elementary schools, 28.6 % at junior high schools, 11.1 % at senior high schools, and 4.3 % at special needs schools. Of the schools, 26.3 % had 201–400 students, 20.5 % had 401–600, and 18.2 % had 61–200. About half (53.7 %) of the *Yogo* teachers had 20 years or more of experience. Most (92.0 %) of them did not have nursing experience. Most of them reported that they “know roughly” about AN, BN and BED (70.7 %, 69.9 % and 55.5 %, respectively), while most reported that they “do not know well” about ARFID and Others (58.8 % and 58.9 %, respectively).

**Table 1:** Attributes of Yogo teachers (*n* = 1,886)

		*n*	%
Location	Government Ordinance Designated City	344	19.4
	Other Area	1428	80.6
	Missing	114	-
School Type	Elementary school	1037	55.8
	Junior high school	529	28.4
	Senior high school	206	11.1
	Special needs school	80	4.3
	Missing	26	-
Number of Students	1–60	160	8.7
	61–200	333	18.2
	201–400	481	26.3
	401–600	375	20.5
	601–800	236	12.9
	801–1000	193	10.5
	1001+	54	2.9
	Missing	54	-
Years of Experience	1–5	335	18.0
	6–10	227	12.2
	11–20	299	16.1
	20–38	999	53.7
	Missing	26	-
Nursing Experience	Experienced	149	8.0
	Not experienced	1718	92.0
	Missing	19	-

Note: % was calculated without missing values (effective %)

**Table 2:** Amount of ED knowledge of Yogo teachers (*n* = 1,886)

	AN		BN		BED		ARFID		Others	
Amount of Knowledge	*n*	%	*n*	%	*n*	%	*n*	%	*n*	%
Know well	206	15.0	139	10.2	75	5.5	26	1.9	11	1.0
Know roughly	968	70.7	955	69.9	755	55.5	325	23.9	165	15.0
Do not know well	184	13.4	261	19.1	502	36.9	800	58.8	647	58.9
Do not know anything	11	0.8	12	0.9	28	2.1	209	15.4	275	25.0
Missing	517	-	519	-	526	-	526	-	788	-

### Statistical analysis 1: encounter rates for each ED type

The results of the four prefecture surveys were combined, and the encounter rates (the proportion of *Yogo* teachers who had encountered students with an ED) by ED type were calculated to be (Table 3, Fig. 2):

$$ \mathrm{AN}\ \left(45.1\ \%\right) > \mathrm{B}\mathrm{N}\ \left(14.5\ \%\right) > \mathrm{ARFID}\ \left(13.0\ \%\right) > \mathrm{B}\mathrm{ED}\ \left(7.8\ \%\right) > \mathrm{Others}\ \left(5.4\ \%\right). $$

**Table 3:** Encounter rates for each ED type

ED Type	*n*	Encounter rate (95 % CI)
AN	850	0.451 (0.428–0.473)
BN	274	0.145 (0.129–0.161)
BED	148	0.078 (0.066–0.091)
ARFID	246	0.130 (0.115–0.146)
Others	102	0.054 (0.044–0.064)

Note: *N* = 1,886, *n* = number of Yogo teachers who encountered students with this type of ED

### Statistical analysis 2: factors affecting the encounter rate (by ED type)

Table 4 shows the results of the logistic analyses, which were used to verify the factors affecting the encounter rate. The significant factors for AN were location (OR = 1.51), school type (OR = 2.61 ~ 6.56), number of students (OR = 1.00), years of experience (OR = 1.05), and knowledge of AN (OR = 2.59). Those for BN were school type (OR = 3.62 ~ 15.2), years of experience (OR = 1.04), and knowledge of BN (OR = 2.84). Those for BED were school type (OR = 3.03 ~ 12.8), years of experience (OR = 1.05), and knowledge of BED (OR = 3.35). Those for ARFID were location (OR = 1.68), years of experience (OR = 1.03), and knowledge of ARFID (OR = 4.23). Those for Others were school type (OR = 9.21), years of experience (OR = 1.03), and knowledge of Others (OR = 3.81).

**Table 4:** Factors affecting the encounter rate (By ED type)

		AN (*n* = 850)		BN (*n* = 274)		BED (*n* = 148)		ARFID (*n* = 246)		Others (*n* = 102)	
Factor	Level	n0/n1 (%)	*OR*	n0/n1 (%)	*OR*	n0/n1 (%)	*OR*	n0/n1 (%)	*OR*	n0/n1 (%)	*OR*
Location	Other Area	606/1325 (45.7 %)	1.00	209/1208 (17.3 %)	1.00	106/1132 (9.4 %)	1.00	173/1063 (16.3 %)	1.00	73/927 (7.9 %)	1.00
	Government Ordinance Designated City	192/329 (58.4 %)	1.51*	53/273 (19.4 %)	1.19	28/239 (11.7 %)	1.20	63/241 (26.1 %)	1.68*	26/202 (12.9 %)	1.55
School Type	Elementary school	309/964 (32.1 %)	1.00	65/911 (7.1 %)	1.00	39/870 (4.5 %)	1.00	130/844 (15.4 %)	1.00	45/722 (6.2 %)	1.00
	Junior high school	337/494 (68.2 %)	4.42***	88/386 (22.8 %)	3.62***	49/361 (13.6 %)	3.03***	74/334 (22.2 %)	1.46	31/293 (10.6 %)	1.43
	Senior high school	156/196 (79.6 %)	6.56***	98/172 (57.0 %)	15.2***	46/134 (34.3 %)	12.8***	31/121 (25.6 %)	1.82	7/93 (7.5 %)	1.00
	Special needs school	34/75 (45.3 %)	2.61**	17/69 (24.6 %)	6.79***	11/65 (16.9 %)	8.63***	8/61 (13.1 %)	1.03	18/58 (31.0 %)	9.21***
Number of Students	-		1.00***		1.00		1.00		1.00		1.00
Years of Experience	-		1.05***		1.04***		1.05***		1.03**		1.03*
Nursing Experience	no	771/1601 (48.2 %)	1.00	239/1428 (16.7 %)	1.00	130/1333 (9.8 %)	1.00	231/1271 (18.2 %)	1.00	90/1086 (8.3 %)	1.00
	yes	74/140 (52.9 %)	0.86	31/121 (25.6 %)	0.95	17/106 (16.0 %)	1.11	14/97 (14.4 %)	0.85	11/87 (12.6 %)	0.74
Knowledge (ED)	1–4		2.59***		2.84***		3.35***		4.23***		3.81***

Note: *p < .05, **p < .01, ***p < .001. OR: Odds Ratio. n0/n1 = number of Yogo teachers who encountered ED/number of Yogo teachers = encounter rate Response variable = experience encountering ED students for each ED type (yes/no)

### Discussion

In this study, to improve the way *Yogo* teachers are supported in their efforts to provide early support to ED students, the encounter rates of EDs in a wide area of Japan were calculated and factors that could affect those rates were examined.

### Statistical Analysis 1: Encounter rates for each ED type

The order of the encounter rates for all four school types combined was AN > BN > ARFID > BED > Others. Therefore, the order of the encounter rates for seven- to 18-year-old students (in elementary/junior high/senior high/special needs schools) was believed to be the same. (Note that the order of BN and ARFID might be reversed because their confidence intervals overlapped (BN [0.129, 0.161], ARFID [0.115, 0.146]). In that case, the order would be AN > ARFID > BN > BED > Others).

In the comparison of our results with those of surveys done outside Japan, please note that although encounter and prevalence rates are different, no encounter rates are available from outside Japan, so we used the prevalence rate for comparison. American students aged 13–18 were interviewed face to face, and the order of prevalence rates was found to be BED > BN > AN [24]. An English medical institute surveyed children aged 10–19, and the order was found to be EDNOS > AN > BN (EDNOS: Eating Disorder Not Otherwise Specified) [20]. It was reported in a number of previous studies that the onset rates of EDNOS and BED in western Europe had increased in those years, while those of AN and BN in the United States and Europe had been constant or decreased since 1970 [20, 22, 35].

In contrast, a survey of *Yogo* teachers [18] regarding high school girl students found the order of prevalence rates to be AN > EDNOS > BN. Although the survey methods of these studies were different from ours and we cannot definitively draw any conclusions, AN seems to be the most prevalent ED in Japan. Thus, it would be effective to provide support for AN patients here.

### Statistical Analysis 2: Factors affecting the encounter rate (by ED type)

The first finding was that the factors affecting the encounter rates for all ED types were years of experience and knowledge of ED. The OR of years of experience ranged from 1.03–1.05, which means that the rate increases 1.03-1.05 times as the years of experience increase by one year. Although this result can be interpreted as “*Yogo* teachers enhance their skill in finding students with EDs as their experience increases,” a simpler explanation is also possible, that “the probability of encountering students with EDs increases as *Yogo* teachers work longer.” Actually, a survey of *Yogo* teachers who had worked at senior high schools for more than 10 years reported that the more their years of experience, the more various types of EDs they encountered.

Secondly, the relatively high ORs (2.59-4.23) implied that the amount of knowledge affected the encounters with students with an ED. It was also reported that the proportion of emaciation (extreme thinness) decreased after *Yogo* teachers were supplied with training on ED prevention. These facts imply that it is necessary to offer ED training in schools to improve the early support of students with EDs.

In particular, our results showed that the ORs of ARFID and Others were over 3.5. For ARFID, the ORs of all of the school types were not significant, which means that the encounter rates of all school types did not differ much and which coincided with studies reporting that ARFID is distributed among pre-puberty children as well as very young children [36]. Thus, it can be concluded that it is necessary to educate *Yogo* teachers about ED in all school types.

On the other hand, for Others, only the OR of special needs schools was significant. Some studies reported cases in which pica- and rumination-related obstacles (which were included in Others) co-existed with developmental disorders [37, 38]. As special needs schools are schools for educating children with disorders, it makes sense that the encounter rate for Others is high in that school type. Thus, it can be inferred that it would be effective to support *Yogo* teachers at special needs schools in their provision of early support for Others students.

Thirdly, the factor that did not affect the encounter rate was “nurse experience.” One survey of *Yogo* teachers with and without nursing licenses reported that personal effort contributed to the gathering and learning of ED information for both groups of teachers. These findings imply that nursing experience is not required to successfully provide early support for students with EDs.

For AN and ARFID, the ORs of schools in government-ordinance-designated cities ranged from 1.51–1.68, which means that the encounter rates were higher in government-ordinance-designated cities than in other areas. Because government-ordinance-designated cities can be roughly seen as urban areas and other areas as rural areas, this result was different from those of previous studies showing that the prevalence rates of urban and rural areas were almost the same [17, 18, 24, 39–41]. The use of criteria based on DSM-5 rather than DSM-IV, which was used in the previous studies, might explain this discrepancy. For example, there could have been a considerable number of students with AN, BED, or ARFID who could not be classified into the corresponding categories because of the strictness of the classification criteria of DSM-IV, but because our criteria were based on DSM-5 such students were classified correctly, making our encounter rates higher.

For school type, the ORs of all ED types except ARFID were significant. The ORs of senior high schools (with students aged from 15–18) were high (6.56–15.2) for AN, BN, and BED (the ORs of BN and BED were over 10), which coincided with the results of previous studies. An English survey [20] reported that the prevalence rates of 15- to 19-year-old children were higher than those of 10- to 14-year-old children for AN, BN, and EDNOS. An American survey [24] also reported that the prevalence rates for BN of 15- to 16- and 17- to 18-year-old children were higher than that of 13- to 14-year-old children. These findings imply that it is necessary to support BN and BED in senior high schools. In comparison, the encounter rates in junior high schools were lower, but still significant (3.03-4.42). In particular, the OR of AN was high at 4.42, which implies that we should support AN beginning in junior high school.

For the number of students, only the OR of AN was significant at 1.001, which means that *Yogo* teachers at larger schools were more likely to encounter a student with AN. Generally speaking, teachers would be more likely to encounter various types of ED as the number of students increases. However, our results indicate that the number of students is not a significant predictor for any of the ED types except AN. The difference in the sample sizes may explain this, because the sample size of AN (850) was much bigger than those of ARFID and Others (246 and 102, respectively). The fact that the ORs of these three types were the same (1.001) also supports this explanation. (Note that the larger the sample size, the more likely it is that the OR becomes significant.)

These findings imply that it would be effective to offer ED knowledge to *Yogo* teachers mainly at school types with high encounter rates in order to enhance the early support of ED students.

Finally, we discuss the value of using DSM-5. In Japanese schools, a number of students have been reported to avoid school lunch, eat from their own lunch boxes, or eat their own hair (or things that are not considered edible). This suggests that ARFID and Pica are prevalent, and it would be effective to monitor the health condition of students based on the DSM-5 criteria. In fact, descriptions of students with ARFID or Pica were common in the free-writing section of our questionnaire, in all prefectures. Therefore, a questionnaire survey based on DSM-5 is believed to be appropriate for clarifying the actual ED situation in schools in order to assist *Yogo* teachers in their support of students with EDs.

### Limitations and recommendations

One of limitations of our study is that a diagnosis by a *Yogo* teacher may be less reliable than one by a doctor. Although we understand this limitation, we used the diagnosis by the *Yogo* teachers for two reasons:

1. In Japan, because doctors are allowed to enter schools for school health checkups just once a year, it is difficult for them to know the daily health condition of the students. In comparison, *Yogo* teachers stay in the schools and witness the health condition of students on a daily basis. Moreover, they are able to obtain information about students from other teachers or students. Thus, diagnosis by *Yogo* teachers may to some extent be more accurate than that by a doctor.
2. Because Japanese students with EDs seldom go to medical institutions by themselves, *Yogo* teachers play an important role in finding them and supporting them. For this purpose, it is necessary to offer *Yogo* teachers information that is useful for supporting ED students, and the encounter rate is one such piece of information. Because our ultimate objective is to support *Yogo* teachers, we consider the subjective encounter rate to be more suitable than the objective prevalence rate, and we considered the encounter rates reported by *Yogo* teachers to be more suitable than those of doctors.

However, our choice created another limitation – a lack of prevalence rates. What we surveyed was the encounter rate and not the prevalence rate. That is, what we obtained was “the proportion of *Yogo* teachers who had encountered EDs” and not “the proportion of students with EDs.” For example, there are students with EDs who are not found by *Yogo* teachers because the teachers do not have sufficient knowledge or the students avoid visiting the offices of the *Yogo* teachers. Thus, the encounter rate differs from the prevalence rate.

The prevalence rate is also useful information, and should be determined in a future study by asking students directly if they have an ED. In such a study, information such as the age and sex of the students should also be obtained, as it could contribute to better support for *Yogo* teachers in their early assistance for students with EDs.

Other limitations include the questionnaire distribution methods. In our survey, the questionnaire was distributed by hand at a seminar hall or by mail. This difference in methods of distribution might have led to a difference in the degree of self-exposure to the questionnaire. A single survey method should be used in the future. In addition, our sample might be biased, as most of the surveys were collected from outside government-ordinance-designated city areas, which are usually rural areas. We will need to gather samples equally from inside and outside government-ordinance-designated cities in our future study.

## Conclusions

In this study, questionnaire survey was administered to *Yogo* teachers at elementary/junior high/senior high/special needs schools in a wide area of Japan regarding their rates of encounter with students having various types of ED (based on DSM-5). The encounter rates were then calculated, and factors affecting the encounter rate were examined based on the survey results. The results showed the order of encounter rates to be AN > BN > ARFID > BED > Others for students aged 7–18 (elementary/junior high/senior high/special needs schools). Because AN was found at a significantly higher rate than the other types of ED, our study implies that supporting AN would be effective.

One factor affecting the encounter rates of all ED types was ED knowledge. Moreover, *Yogo* teachers at senior high schools were most likely to encounter students with AN, BN, or BED and those at special needs schools were most likely to encounter students with Others. These findings imply that in order to identify and support students with EDs early, it is necessary to offer knowledge regarding the ED type with the highest encounter rate by school type.

## Abbreviations

AN: anorexia nervosa
ARFID: avoidant/restrictive food intake disorder
BED: binge eating disorder
BN: bulimia nervosa
CI: confidence interval
ED: eating disorder
EDNOS: Eating disorder not otherwise specified
EOED: early onset EDs
Others: pica rumination disorder, etc
